# In Tribute to Michael Goodin

**DOI:** 10.3390/v13010078

**Published:** 2021-01-08

**Authors:** Jeanmarie Verchot, Andrew O. Jackson, Anne E. Simon

**Affiliations:** 1 Department of Plant Pathology and Microbiology, Texas A&M University, College Station, TX 77802, USA; 2 Department of Plant and Microbiology, University of California, Berkeley, CA 94720, USA; andyoj@berkeley.edu; 3 Cell Biology and Molecular Genetics, University of Maryland, College Park, MD 20742, USA; simona@umd.edu

It is with great sadness and sympathy for his family and the plant virology community that we convey the passing of Michael Goodin unexpectedly in December 2020. Michael contributed enormously as co-editor of this special issue on *Emerging Plant Viruses* for MDPI-Viruses; hence, we wish to dedicate this issue to his memory. We are calling this issue a passion project in memory of Michael, because, if you ever had the honor to work with him or interact with him at a study section or conference, you know of his exuberance and passion for plant virology. Michael’s well-respected research focused mainly on plant-infecting rhabdoviruses and the biochemistry and cell biology of virus/host interactions during infection. His love for coffee led to his recent work with coffee ringspot viruses, which are emerging threats to coffee production and quality. But Michael was much more than an accomplished scientist. He had a special passion for his family, friends, and colleagues. He was passionate about photography and public and science education. He enjoyed travel, particularly in national and state parks, as well as abroad, as illustrated by his outstanding photographs. Michael loved to talk about growing up in Jamaica and the deliciousness of Jamaican cuisine; he even brought goat to cook an unforgettable, authentic Caribbean meal after giving an invited seminar at the University of Maryland! 


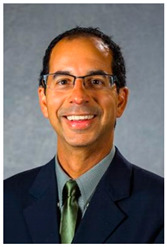


Dr. Michael Goodin (1967–2020)

Michael obtained a PhD in 1995 from Pennsylvania State University in Pete Romaine’s lab. There, he generated a productive thesis that investigated the virus-induced La France disease in cultivated mushrooms. He then spent five years working as a postdoc in Andy Jackson’s lab at UC-Berkeley. Michael obtained a faculty position in the Plant Pathology Department at the University of Kentucky in 2002, and was a professor and the Director of the Plant Science Biological Imaging Facility.

In summary, Michael was a wonderful friend and colleague to so many, and an enthusiastic supporter of plant virology. He was vibrant, creative, and a passionate lover of life. We hope that when you read this special issue, and the letter from Michael, you share with us the memory of his desire to spread the word about emerging viruses that can impact the foods we eat (and drink). We hope that those of you who knew Michael Goodin will reflect on his research contributions to plant virology, the sense of community he instilled amongst us, and his inspiration to students and colleagues, and smile when remembering his laughter and joy at conferences. 

Thank you Michael, for all that you have done. You will be greatly missed.

